# Analysis of BlaEC family class C beta-lactamase

**DOI:** 10.1093/femsle/fnad097

**Published:** 2023-09-26

**Authors:** Jiri Schmidt, Veronika Zdarska, Milan Kolar, Patrik Mlynarcik

**Affiliations:** Department of Biotechnology, Faculty of Science, Palacky University Olomouc, 17. listopadu 1192/12, 779 00 Olomouc, Czechia; Department of Microbiology, Faculty of Medicine and Dentistry, Palacky University Olomouc, Hnevotinska 3, 779 00 Olomouc, Czechia; Department of Microbiology, Faculty of Medicine and Dentistry, Palacky University Olomouc, Hnevotinska 3, 779 00 Olomouc, Czechia; Department of Microbiology, Faculty of Medicine and Dentistry, Palacky University Olomouc, Hnevotinska 3, 779 00 Olomouc, Czechia

**Keywords:** AmpC beta-lactamase, antibiotic resistance, mutation, PCR, primer

## Abstract

Recent years have witnessed an increased prevalence of intrinsic and acquired beta-lactamase-producing bacteria, severely limiting human and veterinary medicine therapeutic options. The present study aimed to design specific oligonucleotides for rapid PCR detection of the cephalosporinase-encoding gene *blaEC* (BlaEC family class C beta-lactamase). A total of three primers were designed to detect 2281 variants of the *blaEC* gene and two sets of primer pairs were also tested against DNA from 11 strains. The study indicates that the proposed primers should be able to detect 100% of all described *blaEC* genes in different bacterial strains and monitor their spread. After comparing the amino acid sequences, a phylogenetic tree was created based on the presence of conserved amino acids and homologous motifs. More than 24 760 mutations in BlaEC enzymes have been identified. The mutations involving 371 amino acid positions and these hotspots can change the structure and activity of the monitored enzymes. We predicted several BlaEC enzymes with a broadened substrate activity against higher-generation cephalosporins.

## Teaser

Comprehensive information on BlaEC family class C beta-lactamase and their detection by PCR.

## Introduction

An influential group of enzymes that allow bacteria to resist the effects of beta-lactam antibiotics are beta-lactamases (*bla*) of the AmpC type. These are cephalosporinases that hydrolyze most penicillins, cephalosporins, oxyimino-cephalosporins, and monobactams. These antibiotic groups contain frequently applied antibiotics, and the resistance of bacterial pathogens to their effect represents a serious clinical problem. In the case of continuous production of the mentioned enzymes, the minimum inhibitory concentration (MIC) values are increased above the clinical breakpoints of the respective antibiotics. Another problem is the possibility of switching from inducible to continuous production of the enzymes during antibiotic administration, which can lead to treatment failure and, in the case of sepsis, to the death of the patient. Classic beta-lactamase inhibitors (clavulanic acid and sulbactam) have zero effect in the case of this group.

AmpC beta-lactamases encoded by chromosomal genes are widely distributed in most bacterial members of the order *Enterobacterales* as well as bacteria from other families, such as *Acinetobacter* spp., *Aeromonas* spp., or *Pseudomonas* spp. In many bacteria, chromosomal *ampC* gene expression is usually low. The expression is induced at the time of response to an external stimulus, such as a beta-lactam antibiotic. Individual beta-lactam antibiotics differ in their ability to induce AmpC expression, with strong inducers including penicillins and first-generation cephalosporins (Jacoby 2009). Beta-lactamase inhibitors also act as inducers, especially clavulanic acid (Weber and Sanders 1990). Further, AmpR, AmpD, and AmpG (cell wall metabolism proteins) are involved in the induction of the *ampC* gene (Hanson and Sanders 1999). The cytosolic protein AmpD, which acts as a negative expression regulator, is also involved in regulating AmpC expression (Jacobs et al. 1995).

According to the Ambler classification scheme, these beta-lactamases are classified in class C and, according to the Bush–Jacoby–Medeiros classification, they belong to group 1 (Bush 2013, Bush and Jacoby 2010, Harris and Ferguson 2012). The new serine enzymes of subgroup 1e are variants of group 1 with greater activity against ceftazidime and other oxyimino-lactams due to amino acid substitutions, insertions or deletions. They have been designated as extended-spectrum AmpC (ESAC).

Representatives of ESAC are, e.g. some types of *Acinetobacter*-derived cephalosporinases (ADCs) (Naas et al. 2017) and BlaEC subtypes, as described below. Some ESAC also hydrolyze carbapenems. These include the chromosomally encoded ADC-68 enzyme described in *Acinetobacter baumannii* (Jeon et al. 2014). In addition, weak hydrolytic activity against carbapenems has also been reported for some other class C beta-lactamase subtypes such as ACT-28 and CMY-10 (Bonnin et al. 2020).

Regarding BlaEC AmpC beta-lactamases (primarily assigned to the genus *Escherichia*), a total of 2281 different subtypes in the genera *Escherichia* and *Shigella* (2061 subtypes in the genus *Escherichia* and 137 subtypes in the genus *Shigella*) have been identified so far based on the BLDB database (Naas et al. 2017). This group of beta-lactamases is mainly chromosomally encoded with a narrower hydrolytic spectrum. However, some BlaEC variants have been found to cause resistance to extended-spectrum cephalosporins, with significantly increased MICs for ceftazidime and cefepime. This extended-spectrum hydrolysis has been reported for different BlaEC subtypes (Mammeri et al. 2006), including BlaEC-7 (GenPept accession number AAD28043), BlaEC-8 (AAZ85974), BlaEC-9 (AAZ42369), BlaEC-10 (AAZ85975), BlaEC-11 (AAT07063), and BlaEC-12 (AAT07064) (numbering according to the BLDB). Moreover, Doi et al. (2004) described a unique BlaEC-2 (BAC99094) AmpC enzyme in a clinical isolate of *Escherichia coli*, in which a tripeptide deletion (Gly286–Ser287–Asp288) was responsible for resistance to cephalosporins including oxyimino-cephalosporins.

The main objectives of our study were to design specific oligonucleotides for rapid PCR detection and to monitor the distribution of all previously described BlaEC enzymes among bacteria. Last but not least, we aim to provide more comprehensive information about this group of enzymes.

## Materials and methods

### Bacterial strains of animal origin

In total, 11 *blaEC*-positive *Escherichia* strains of animal origin from the bacterial collection at the Department of Microbiology, Faculty of Medicine and Dentistry, Palacky University Olomouc were used as positive controls—*blaEC-41 E. coli* (GenBank accession number JAMXZX000000000); *blaEC-73 E. coli* (JAMXZV000000000); *blaEC-126 E. coli* (JAMXYQ000000000); *blaEC-134 E. coli* (JAMXZO000000000); *blaEC-135 E. coli* (JAMXZY000000000); *blaEC-193 E. coli* (JAMYAA000000000); *blaEC-813 E. coli* (JAMXZM000000000); *blaEC-1149 E. coli* (JAMYAK000000000); *blaEC-1861 E. coli* (JAMXZI000000000); *blaEC-like Escherichia fergusonii* (JAMXYS000000000); and *blaEC-like E. fergusonii* (JAMXZU000000000). More details about the *Escherichia* isolates used in the study are listed in Table S1 (Supporting Information).

### Primer design and *in silico* analysis of BlaEC sequences

A total of 2281 sequences of genes encoding BlaEC enzymes, containing only the coding regions without their promoters, described in the BLDB database (last accessed on 2 May 2023) (Naas et al. 2017) were downloaded from the GenBank database (http://www.ncbi.nlm.nih.gov/genbank). The bioinformatics software Geneious Prime 2023.2.1 (Biomatters, New Zealand) (Kearse et al. 2012) was used to compare nucleotide and amino acid sequences. The *blaEC* nucleotide sequences and their amino acid sequences were aligned using MUSCLE algorithm (Edgar 2004) and Geneious alignment (default settings in both) as implemented in Geneious Prime, respectively. Protein statistics generated in Geneious within the sequence alignments at the Cost Matrix of Blosum62 were used to study amino acid substitutions.

Primers were designed as described previously (Mlynarcik et al. 2021b).

### Mutation frequency studies

The mutation frequency for a given site is defined as the number of sequences that have a mutation (a different amino acid listed in the BlaEC-1 reference sequence) on that site. The numbering of the amino acid residues is by the structural alignment-based numbering of class C beta-lactamases or the SANC scheme (Mack et al. 2020).

### Testing specificity of designed primers with *in silico* primer binding tests and conventional PCR

*In silico* binding tests were performed to evaluate the specificity of three primer pairs. All primers were tested against *blaEC* nucleotide sequences using the Test with Saved Primers option in Geneious. The specificity of BlaEC-F1/R1 and BlaEC-F2/R2 primers was also tested with conventional PCR using genomic DNA extracted from *E. coli* isolates. For the preparation of the reaction mixture (25 µl), we used Combi PPP Master Mix (Top-Bio Prague, Czech Republic) as described by the manufacturer. The PCR samples were examined using a 1% agarose gel. The gel was treated with SYBR Safe dye from Invitrogen and observed under a UV transilluminator for visualization.

### Phylogenetic tree construction

A maximum-likelihood phylogenetic tree was generated and visualized using the protein alignment with PhyML (Guindon et al. 2010) implemented within Geneious Prime with the Le-Gascuel substitution model and without bootstrapping.

## Results

**Figure 1.**
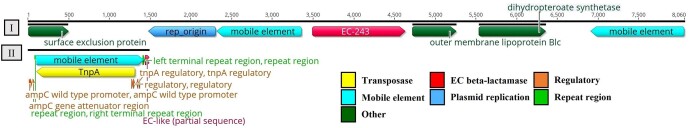
Genetic structure surrounding the *blaEC* genes. The map was carried out by Geneious Prime. I—JN412137: 9007 bp (*E. coli* strain 108 plasmid pT108; linear view); II—AY559027: 1486 bp (*E. coli* insertion sequence IS10 TnpA).

Our study attempted to design primers to detect 2281 variants of *blaEC* genes found in *Escherichia* and *Shigella* strains based on the BLDB database. Interestingly, a further BLASTn search of the *blaEC* genes against the NCBI database identified a series of enterobacteria carrying the same beta-lactamase type (e.g. class C beta-lactamase BlaEC-5 in *Enterobacter hormaechei*—CP056649, BlaEC-1 in *Salmonella* spp.—CP046033), including the described *blaEC-1-* and *blaEC-243*-harboring plasmid in *Klebsiella oxytoca* (CP069925) and *E. coli* (JN412137), respectively. Interestingly, in the latter plasmid, we found that two mobile elements, including the insertion sequence IS*10* (upstream) and IS*CR2* (downstream), were located in the vicinity of the *blaEC-243* (Fig. 1-I). In addition, in another *E. coli* strain (AY559027), IS*10* flanked by the 9-bp direct repeat sequences (CGTTTTGTA) were inserted between the *ampC* attenuator region and the start codon of the partial *blaEC-like* sequence (Fig. 1-II).

A total of three specific primer pairs were created (Table 1) using Primer3 (Geneious). The primer-BLAST results showed that these oligonucleotides could detect all allelic variants of this beta-lactamase type. Briefly, the BlaEC-F1/R1 primers described earlier (Mlynarcik et al. 2021a) could capture 206 specific *blaEC* variants. Another 139 specific variants could be tested using the BlaEC-F2/R2 primers. In addition, both primer pairs can jointly detect another 1927 BlaEC variants, corresponding to amplified PCR products, using DNA from tested isolates, as described below. The remaining nine distinct subtypes of BlaEC enzymes could be verified using the specific BlaEC-F3/R3 primers. The specificity of the first two primer pairs was also analyzed by means of PCR analysis. PCR performed on DNA from *Escherichia* isolates using BlaEC-F1/R1 and BlaEC-F2/R2 primer sets produced DNA fragments of the expected sizes (307 and 761 bp; Figure S1, Supporting Information) and confirmed the target specificity of the primer pairs.

**Table 1.** Primer sequences used to detect the *bla*_EC_ genes by PCR.

Primer name	Sequence (5′–3′ direction)^a^	Target (variants)	Amplicon size (bp)	Tm (°C)	Reference
BlaEC-F1/R1	KAAATCCTCAAGCGACKTGC, AATGGTCGACTTYACACCA	206 specific BlaEC variants (BlaEC-1/-42/-74/-100/-101/-104/-122/-123/-144/-169/-182/-188/-211/-250/-266/-287/-289/-330/-331/-336/-337/-343/-372/-400/-401/-437/-443/-451/-452/-460/-492/-547/-548/-559/-659/-671/-706 to -709/-728/-742/-786/-791/-816/-859 to -861/-892/-899/-928/-979/-1041/-1047/-1056/-1059/-1077/-1079/-1081/-1100/-1101/-1110/-1115/-1116/-1126/-1144/-1145/-1201/-1202/-1211/-1339/-1367/-1387/-1410/-1433/-1440/-1489/-1497/-1520/-1523/-1546/-1601/-1631/-1672/-1673/-1683/-1785 to -1787/-1796/-1852/-1879/-1881/-1897/-1923 to -1925/-1927 to -1931/-1933/-1948/-1995/-1996/-2008/-2049/-2082/-2101 to -2103/-2105/-2106/-2108 to -2120/-2122/-2125/-2140/-2142 to -2155/-2159 to -2176/-2178 to -2187/-2189 to -2199/-P1 to -P4/-P6/-P9 to -P11/-P48 to -P50/-P69 to -P73/-P75 to -P81) and many others*	307	55	Mlynarcik et al. (2021a)
BlaEC-F2/R2	ACAAAATACTGGCCTGAACT, TTTTTGTTAGCCAGCATCAC	139 specific BlaEC variants (BlaEC-14/-17/-18/-40/-43/-44/-47/-49/-60/-71/-75/-107/-108/-116/-117/-132/-142/-151 to -153/-155/-163/-203/-241 to -243/-248/-252/-253/-275/-286/-298/-310/-326/-367/-377 to -379/-381 to -388/-402 to -415/-417 to -419/-421 to -423/-426/-427/-438/-446/-447/-467/-468/-471/-473 to -475/-484/-485/-488/-489/-519/-639/-658/-669/-754 to -767/-769 to -773/-776/-777/-792/-896/-989/-1102/-1104/-1130/-1205/-1227/-1292/-1293/-1370/-1446/-1449/-1485/-1593/-1595/-1596/-1615/-1676/-1684/-1818/-1820/-1830–1832/-1912/-1957/-1993/-2015/-2043/-2044/-2077/-2100/-2107) and many others*	761	52	This study
BlaEC-F3/R3	TACTTTACCTGGGGCTATGC, CTTCAGCATCTAACGCCC	BlaEC (9 variants: BlaEC-380/-416/-420/-768/-774/-775/-1908/-2177/-2188)	541	55	This study

a For degenerate primers: K = G or T; Y = C or T.

* Primer pairs can detect an additional 1927 BlaEC variants.

In addition, a point mutation study showed that 24 766 amino acid changes were detected in BlaEC enzymes. The most common amino acid changes recorded were as follows: A → T (1807 times), D → A (1543 times), T → A (1478 times), E → D (1477 times), and K → N (1388 times). By contrast, amino acid changes recorded only once were found in 21 cases, such as A → C or F or L, D → C or P or S, E → N or Y, F → K, G → Q, and I → R. More detailed information about point mutations in BlaEC enzymes is given in Table S2 (Supporting Information).

**Figure 2.**
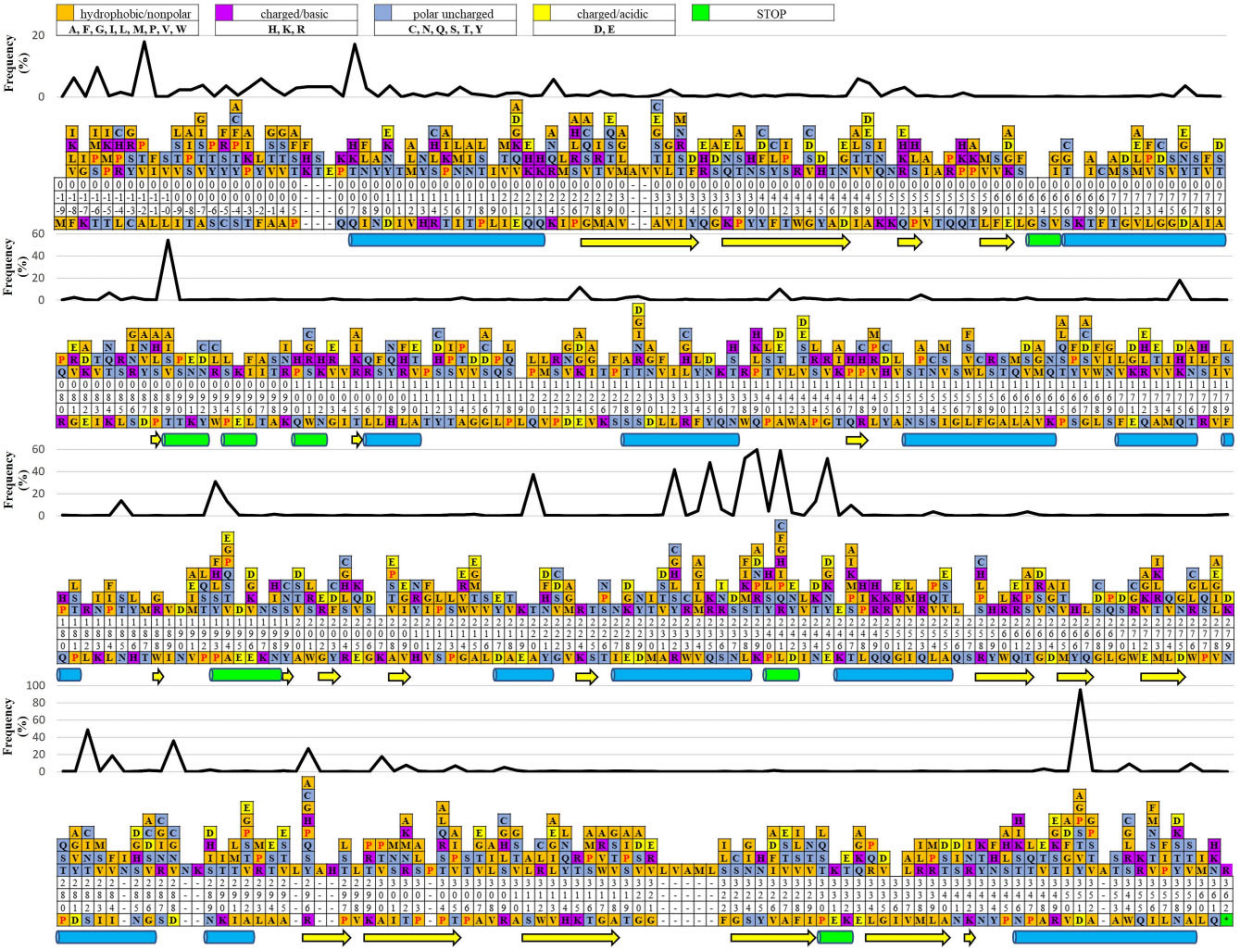
Amino acid changes along the primary sequence of BlaEC enzymes, shown with evolutionary conservation. The observed amino acid substitutions in 2280 BlaEC enzyme variants are above the BlaEC-1 enzyme sequence. Proline substitutions are highlighted in red. The colored bars characterize the general categories of amino acids. Residues without bars represent zero identified substitutions. Numbers are read vertically and indicate SANC-based amino acid residue positions. Above the observed amino acid substitutions are the change frequencies for each position. Alpha-helices (blue barrel), 3_10-_helix (green barrel), and beta-strand or beta-bridge (yellow arrows).

Figure 2 shows the mutational frequencies for each position, highlighting evolutionary hotspots. It shows many hotspots along multiple sequence alignments covering more than 24 760 mutations, of which 27 hotspots had more than 200 mutations. The frequency of mutations was high at residue positions Asp351, Lys239, Leu241, Thr89, Leu238, Glu245, Ser282, Gln235, Arg232, and Ala220 (2159, 1368, 1350, 1238, 1188, 1184, 1108, 1101, 955, and 854 mutations, respectively) (data not shown). These data suggest that beta-strands mutated less frequently and appear to be more resistant to mutations than helices.

Table 2 illustrates predicted extended-spectrum BlaEC enzymes based on specific amino acid changes involved in the broadening of the hydrolysis spectrum described by Doi et al. (2004) and Mammeri et al. (2006). Based on their findings, we considered a total of four amino acid changes within the entire BlaEC enzyme sequences at positions 287, 296, 298, and 350 (S-287-*, H/R-296-*, V-298-*, and V-350-*) and one deletion of three amino acids (GSD) at positions 286–288 (286-GSD-288); thus, we identified 16 ESAC enzymes. By considering other amino acid changes at these positions, we get a total of 58 ESAC enzymes. Regarding the PCR detection of these 58 ESAC genes, our second primer, designated as BlaEC-F2/R2, can capture all the mentioned variants except *blaEC-861*, which, on the other hand, detects the BlaEC-F1/R1 primer.

**Table 2.** Prediction of extended-spectrum BlaEC enzymes.

Original amino acids	Amino acid change	Positions (according to publications and SANC scheme)	Putative extended-spectrum BlaEC variants
S	**N^a^**	287^**^a^**^	7, 27, 595, 861, 1814
	**C^**^a^**^**		10, 146, 1638, 1922
	G		1046
	I		1237
	R		338, 1236, 1344, 1495
	Deletion		2**^c^**
H/R	**P^a^**	296**^a^**	9, 195, 877
	A		28, 31
	C		1634
	G		1728
	Q		806
	S		40, 43, 44, 47, 49, 60, 75, 117, 132, 140, 142, 155, 298, 438, 473–475, 489
	Y		893
V	**L^**^a^**^**	298**^a^**	8
V	A	350**^a^**	585, 1188
	D		571, 1558, 1719, 1790, 1792
	**F^a^**		11, 12
	G		1716
	I		25, 584, 1189, 1889
GSD	**GSD deletion^b^**	286–288^**^b^**^	2**^c^**

Amino acids highlighted in bold extend the hydrolysis spectrum to include ceftazidime and cefepime according to ^a^Mammeri et al. (2006) and ^b^Doi et al. (2004). ^c^The same BlaEC variant. SANC (structural alignment-based numbering of class C beta-lactamases).

A total of six conserved amino acids (excluding the start codon), glycine and serine at position 63–64 (63-GS-64 in the SANC scheme), lysine at position 67 (K67), leucine at position 119 (L119), serine at position 257 (S257), and isoleucine at position 336 (I336), were identified within the BlaEC enzymes studied (Figure S2, Supporting Information).

Comparison of amino acid sequences of the selected BlaEC enzymes showed 85.8%–99.7% sequence identity between them. For example, BlaEC-68, compared to BlaEC-377 and BlaEC-408, had only 85.8% amino acid identity (54 amino acid differences), and 85.9% identity was found between BlaEC-377 and BlaEC variants P1 and P6, as well as between BlaEC-408 and BlaEC-P6. More detailed information on the BlaEC variants with the lowest amino acid identity is given in Table S3 (Supporting Information). By contrast, for (i) BlaEC-14 and BlaEC-107/-108/-326/-387, (ii) BlaEC-18 and BlaEC-71, (iii) BlaEC-28 and BlaEC-31, (iv) BlaEC-40 and BlaEC-44, (v) BlaEC-47 and BlaEC-49, (vi) BlaEC-75 and BlaEC-117, and (vii) BlaEC-116 and BlaEC-379, 99.7% identity was found (results not shown).

**Figure 3.**
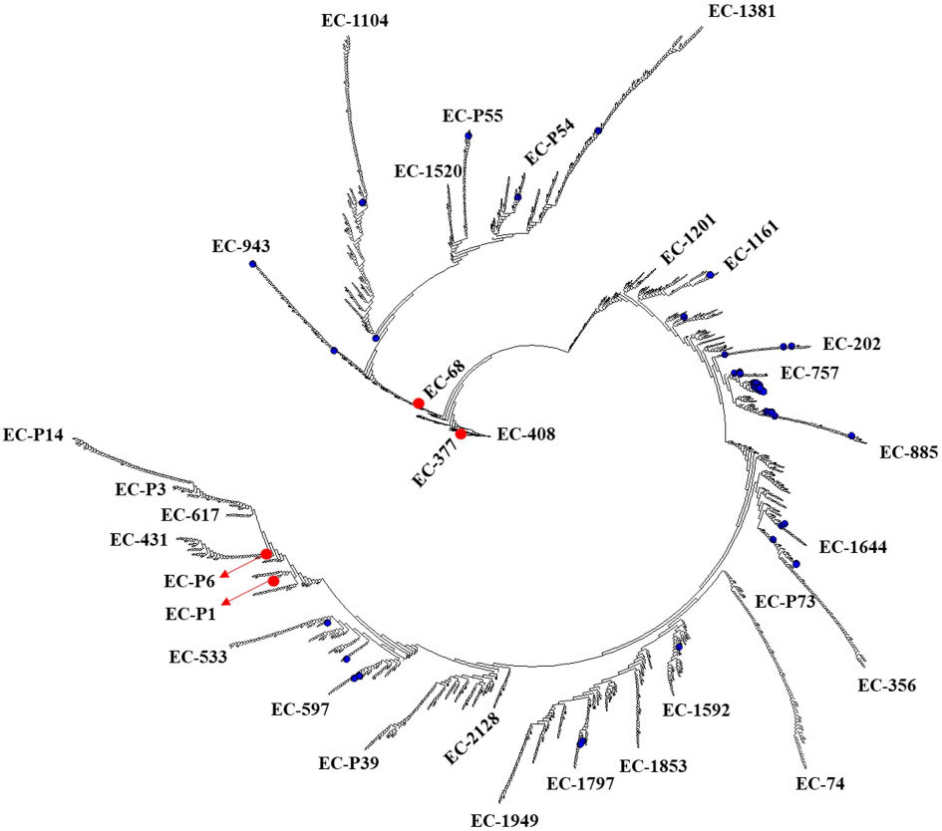
A phylogenetic tree obtained by comparing 2281 BlaEC enzymes using Geneious PhyML. Characteristic BlaEC enzymes are indicated at the end of the branches. Red circles represent some sequence types with the lowest amino acid identity. The 58 putative extended-spectrum BlaEC enzymes are highlighted in blue.

Using a phylogenetic tree, relationships between BlaEC enzymes were illustrated based on the similarity of their amino acid sequences. A rooted phylogenetic tree allowed us to identify several major groups and subgroups (Fig. 3).

## Discussion

According to data from the BLDB database, oxacillinases and the BlaEC most abundant class C beta-lactamases represent more than 48% (3419 variants) of all beta-lactamases described (Naas et al. 2017). Therefore, we focused on this particular type of *ampC* genes. At the same time, Doi et al. (2004) and Mammeri et al. (2006), in their respective studies, detailed seven BlaEC enzyme variants of clinical relevance that show an extended capacity to act on higher-generation cephalosporins. More detailed information about PCR detection of oxacillinases in bacteria is available elsewhere (Mlynarcik et al. 2020).

The cause of bacterial resistance is a mutation in one of the structural or regulatory genes involved in the expression of beta-lactamases of the AmpC type and the subsequent switch from inducible to permanent production. In addition, overproduction can also result from insertions in the AmpC promoter and replacement of the native promoter region with a promoter from other bacteria, ultimately allowing for higher levels of gene expression (Papanicolaou et al. 1990). Furthermore, the horizontal acquisition of AmpC beta-lactamases has recently gained importance and represents an essential driving force in increasing resistance (Mac Aogain et al. 2016). Considering AmpC beta-lactamases of the BlaEC, a large number of these variants have been described, but only some of them are expressed to a sufficient extent to cause antibiotic resistance. For example, as shown in Fig. 1, genes encoding BlaEC production can be expressed using functional promoters represented by insertion sequences, as for oxacillinases (Mlynarcik et al. 2020).

Interestingly, hyperproduction of the AmpC chromosomal enzyme, combined with the loss of outer membrane protein, has been found to play a role in developing carbapenem resistance in *Serratia marcescens* (Suh et al. 2010). An increasing number of papers have described the synergy between AmpC beta-lactamase production, efflux pump overexpression and low outer membrane permeability in clinical isolates. For example, Tomas et al. found that *Pseudomonas aeruginosa* isolates from cystic fibrosis patients that overexpressed *mexA* or *ampC* and reduced *oprD* were associated with beta-lactam resistance (Tomas et al. 2010). Additionally, Liu and colleagues found that low expression of outer membrane porin with cephalosporinase overexpression or extended-spectrum beta-lactamase (ESBL) production and efflux pump overexpression may contribute to carbapenem resistance in carbapenem-insensitive *Enterobacter cloacae* isolates that produce noncarbapenemase in the hospital (Liu et al. 2021). Therefore, detecting all AmpC beta-lactamases, including BlaEC enzymes, is necessary to better understand the development of multidrug resistance.

Recently, we have designed several primers to monitor ESBL genes in clinically significant bacteria (Mlynarcik et al. 2021a,b), focusing on another group of extended-spectrum BlaEC enzymes in this study. Three primers (Table 1) were designed to detect 2281 variants of *blaEC* genes in enterobacteria. However, the first two pairs of primers can detect 2272 variants of the monitored genes, representing more than 99.6% of the total number of subtypes described. Since the plasmid-encoded *blaEC* genes have already been described in enterobacteria, the primers we designed can be used to monitor these resistance genes.

The dataset contained more than 24 760 mutations covering a total of 371 out of 378 residue positions of BlaEC enzymes, here referred to as mutational hotspots. These hotspots had at least one mutation. In particular, the data showed that hotspots located in helices were most frequently mutated (Fig. 2). In addition, proline substitutions are also shown in this figure, as it has been reported in other studies that proline represents the most disruptive substitution (disrupts enzyme function), and methionine is most tolerated (Gray et al. 2017, Vakulenko et al. 2002).

Further, the mutational analysis study suggests that at least 16 subtypes of BlaEC enzymes represent ESAC beta-lactamases taking into account the conclusions made by Doi et al. (2004) and Mammeri et al. (2006). Regarding the PCR detection of these ESAC genes, all these variants could be detected using our first primer, BlaEC-F1/R1. When considering other amino acid changes (Table 2), such as the change from serine to isoleucine or arginine at position 287, the estimated number of ESAC enzymes is 58, but experimental data are needed to support this speculation. The PCR primer F2/R2 reported in this study would allow the detection of 98.3% of ESAC variants (57/58) and would not be able to detect BlaEC-861. This subtype could be verified using a specific BlaEC-F1/R1 primer. In addition, there may be many more of these enzymes. Therefore, we tried to summarize the frequency of all amino acid changes along the entire sequence in all 2281 variants of BlaEC enzymes. The above results could be helpful for scientific personnel working in the field of these beta-lactamases and their drug resistance analysis.

This study highlights the diversity of BlaEC beta-lactamases, and our data may help define the boundaries of the BlaEC enzyme subfamily. On the other hand, these least similar BlaEC variants (Table S3, Supporting Information) may also represent a new type of beta-lactamases. As was the case, e.g. with KLUC-1 and the CTX-M-1 subgroup of beta-lactamases, which shared 85%–86% amino acid identity (Decousser et al. 2001). And the peculiar hydrolysis profile may also be related to this.

PCR and whole genome sequencing (WGS) are valuable tools for screening and monitoring clinically important bacteria producing ESBL and carbapenemases. However, each of these methods has advantages and limitations, and the choice between them depends on the specific objectives and resources of the laboratory or research project.

PCR is particularly cost-effective and helpful in laboratories without access to next-generation sequencing platforms or extensive financial resources. It offers lower cost, faster turnaround time and the ability to detect specific resistance genes, such as *blaEC* genes detected in various enterobacteria and on plasmids. This is critical for understanding the evolution of multidrug resistance in bacteria due to the coexistence and cotransmission of beta-lactamases with other resistance genes. However, PCR is more targeted and, unlike WGS, may not detect other resistance genes or genetic variations not explicitly targeted by the primers used in the PCR assay. On the other hand, WGS requires more resources and capacity but offers a comprehensive analysis of genetic relationships. It enables the detection of all potential resistance genes. It provides a more complete picture of the bacterial genome. It may help better understand the epigenetic mechanisms of phenotypic resistance, enabling clinicians to make rapid clinical decisions that improve patient health outcomes. In addition, it may contribute to the discovery of novel resistance mechanisms. However, WGS can be more expensive, requires more sophisticated equipment and bioinformatics expertise, and takes longer to obtain results. For example, the actual interpretation of the results and the large computer memory needed to process and store the genomic data remain significant challenges.

In summary, our results suggest that the tested primers could be used for PCR to detect and monitor the spread of these genes into other bacterial species. The study also analyzed mutational frequencies, identified evolutionary hotspots and conserved amino acids within the BlaEC enzymes studied, and predicted extended-spectrum BlaEC enzymes based on specific amino acid changes. A phylogenetic tree was used to illustrate relationships between BlaEC enzymes based on their amino acid sequence similarity.

## Supplementary Material

fnad097_Supplemental_File
