# Supplementary material for: Analysis of BlaEC family class C beta-lactamase
Source: FEMS Microbiol Lett. 2023 Sep 26;370:fnad097. doi: 10.1093/femsle/fnad097 (PMC10563145; doi:10.1093/femsle/fnad097)
Supplement: fnad097_Supplemental_File [file fnad097_supplemental_file.docx]

**Table S1.** Genotypic description and antimicrobial susceptibilities of *Escherichia* isolates.

| **Bacterial strains (GenBank accession number)** | **BlaEC beta-lactamases** | **Additional beta-lactamases** | **Antibiotics (mg/L)^a^** | | |
| --- | --- | --- | --- | --- | --- |
|  |  |  | CTX | CTZ | MER |
| *E. coli* CAM213-2 (JAMXZX000000000) | BlaEC-41 | CMY-2, TEM-1 | 16 | 16 | <0.1 |
| *E. coli* S1039-2 (JAMXZV000000000) | BlaEC-73 | CTX-M-15, TEM-1, OXA-1 | 16 | 16 |  |
| *E. coli* CAM47-2 (JAMXYQ000000000) | BlaEC-126 | CTX-M-1 | 16 | 1 |  |
| *E. coli* S 298-1 (JAMXZO000000000) | BlaEC-134 | CTX-M-2 | 16 | 4 |  |
| *E. coli* S1045-2 (JAMXZY000000000) | BlaEC-135 | CTX-M-1 | 16 | 1 |  |
| *E. coli* S 1185-1 (JAMYAA000000000) | BlaEC-193 | CTX-M-1 | 16 | 2 |  |
| *E. coli* S 289-1 (JAMXZM000000000) | BlaEC-813 | CTX-M-1 | 16 | 2 |  |
| *E. coli* S 4443-21 (JAMYAK000000000) | BlaEC-1149 | CTX-M-1 | 16 | 8 |  |
| *E. coli* S 264-3 (JAMXZI000000000) | BlaEC-1861 | CTX-M-1 | 16 | 1 |  |
| *E. fergusonii* CAM44-2 (JAMXYS000000000) | BlaEC-like | CTX-M-1, TEM-1B | 16 | 2 |  |
| *E. fergusonii* S 298-5 (JAMXZU000000000) | BlaEC-like | TEM-1, SHV-12 | 2 | 16 |  |
| Legend: CTX - cefotaxime, CTZ - ceftazidime, and MER - meropenem. ^a^Susceptible (S)/resistant (R) breakpoints (mg/L): CTX, S ≤ 1, R > 2; CTZ, S ≤ 1, R > 4; MER, S ≤ 2, R > 8. | | | | | |

**Table S2.** Point mutations in BlaEC enzymes.

| **Amino acid change** | | **Number of mutations** | **SANC-based amino acid residue positions** |
| --- | --- | --- | --- |
| **from** | **to** |  |  |
| A | C | 1 | 31 |
| A | D | 66 | 49/79/141/143/162/200/231/340 |
| A | E | 150 | 29/31/49/110/141/143/173/194/208/215/255/292/294/295/307/327/348 |
| A | F | 1 | 8 |
| A | G | 15 | -7/-1/4/31/194/215/292/307/310/318/352 |
| A | I | 43 | -7 |
| A | L | 1 | 151 |
| A | M | 3 | 215 |
| A | P | 686 | -12/114/194/208/292/300/352 |
| A | Q | 2 | 29/194 |
| A | S | 139 | -7/-1/4/29/31/49/77/79/98/143/160/162/194/200/208/231/255/292/294/295/310/318/327/340/352/353/359 |
| A | T | 1807 | -12/-7/-1/4/29/31/46/49/77/79/98/110/114/141/143/160/173/194/200/208/215/218/220/231/255/292/294/295/300/307/310/318/327/340/348/352/353/359 |
| A | V | 266 | -12/-7/-1/4/29/31/49/110/114/141/143/151/162/173/194/200/208/215/218/231/255/292/295/300/307/310/318/327/348/352/359 |
| C | F | 6 | -5 |
| C | G | 3 | -13 |
| C | R | 4 | -13/-5 |
| C | S | 3 | -13/-5 |
| C | Y | 77 | -13/-5 |
| D | A | 1543 | 87/130/264/281/351 |
| D | C | 1 | 288 |
| D | E | 196 | 10/47/76/217/242 |
| D | G | 831 | 47/76/123/130/264/275/281/288/351 |
| D | K | 2 | 10 |
| D | N | 47 | 10/47/76/86/123/130/229/242/264/275/288 |
| D | P | 1 | 351 |
| D | S | 1 | 351 |
| D | T | 620 | 351 |
| D | V | 5 | 87/123/281/288/351 |
| D | Y | 11 | 10/76/87/217/229/242/281/351 |
| E | A | 6 | 21/61/82/124/272/333 |
| E | D | 1477 | 21/61/82/124/171/195/196/228/245 |
| E | G | 12 | 21/61/124/171/196/228/245/272/333 |
| E | K | 50 | 21/61/82/95/124/171/196/205/219/228/245/272/331/333 |
| E | N | 1 | 245 |
| E | Q | 4 | 21/205/333 |
| E | V | 5 | 21/196/205/272 |
| E | Y | 1 | 245 |
| F | C | 4 | 41/69/158 |
| F | I | 5 | -18/322/328 |
| F | K | 1 | -18 |
| F | L | 284 | -18/-2/41/134/158/170/179/322/328 |
| F | S | 6 | 60/179/322/328 |
| F | V | 9 | -18/60/170/179/328 |
| F | Y | 3 | -2/41/134 |
| G | A | 8 | 27/36/116/167/286/317/320 |
| G | C | 16 | 27/44/74/116/167/222/268/286/323 |
| G | D | 77 | 36/44/71/74/115/116/167/206/222/263/268/270/286/320/335 |
| G | E | 63 | 81/103/202/214/251/321 |
| G | Q | 1 | 27 |
| G | R | 58 | 81/103/145/159/202/214/251/263/317/321 |
| G | S | 91 | 27/36/44/71/74/75/116/156/159/167/206/222/263/268/270/286/320/323 |
| G | V | 30 | 27/44/75/81/103/115/145/167/214/222/251/263/286/317/320/321/335 |
| G | W | 3 | 214/317 |
| G | * | 2 | 321 |
| H | L | 2 | 186/314 |
| H | N | 4 | 13/108/210/314 |
| H | Q | 2 | 108/314 |
| H | R | 1 | 210 |
| H | Y | 8 | 13/108/186/210/314 |
| I | A | 3 | 48 |
| I | F | 7 | 78/284/356 |
| I | L | 121 | -9/8/16/25/48/243/252/291 |
| I | M | 93 | 16/20/25/33/155/252/283/291/301/356 |
| I | N | 12 | 8/16/33/227/301/329/356 |
| I | R | 1 | 33 |
| I | S | 8 | -9/33/155/283/301/356 |
| I | T | 17 | -9/11/20/33/48/83/291/329/356 |
| I | V | 604 | -9/20/48/78/83/104/155/189/243/252/283/284/301/329/356 |
| K | A | 2 | 24/239 |
| K | D | 12 | 239 |
| K | E | 8 | 37/91/246/332 |
| K | G | 1 | -17 |
| K | I | 8 | -17/50/99/183/290/342 |
| K | N | 1388 | 24/37/50/51/84/91/99/164/183/197/239/342 |
| K | P | 37 | 239/299 |
| K | Q | 28 | 24/37/50/84/164 |
| K | R | 100 | 24/99/224/239/299/315/342 |
| K | T | 170 | 84/126/239/290/299/315/332 |
| L | C | 3 | -14/241 |
| L | F | 156 | -11/62/73/96/107/109/131/157/168/184/238/241/357 |
| L | G | 1 | 241 |
| L | H | 6 | -14/109/241/248 |
| L | I | 44 | -11/19/96/132/184/238/241/248/357/360 |
| L | K | 1 | 238 |
| L | M | 1176 | 59/149/161/238/293/339/360 |
| L | P | 49 | -14/73/117/149/184/241/248/254/269/293/334/339/357 |
| L | Q | 60 | 106/117/161/241/254/269/274/334 |
| L | R | 1176 | -14/85/106/109/149/182/241/293/334/339 |
| L | S | 10 | -10/62/85/107/157/216/238 |
| L | V | 88 | -10/59/73/131/149/157/168/216/254/274 |
| L | W | 2 | 157/168 |
| M | I | 69 | 28/174/230/265/273/338 |
| M | K | 1 | 273 |
| M | L | 8 | 273/338 |
| M | R | 4 | 28/273/338 |
| M | T | 5 | 28/230/265/273 |
| M | V | 5 | 174/265 |
| N | A | 1 | 9 |
| N | D | 31 | 190/237/244/279/285/289/341/358 |
| N | G | 1 | 285 |
| N | H | 37 | 102/137/198/285/289/346 |
| N | I | 5 | 198/289/341/346 |
| N | K | 54 | 102/244/279/343/346/358 |
| N | S | 59 | 137/152/185/198/237/285/289/341/346/358 |
| N | T | 562 | 137/185/244/343/346/358 |
| N | Y | 3 | 9/343/358 |
| P | A | 9 | 5/26/38/88/165/303/304/345 |
| P | F | 11 | 5/193 |
| P | H | 110 | 26/53/88/193/240/345 |
| P | L | 86 | 18/26/38/53/88/94/118/140/144/165/181/192/193/213/240/277/297/303/304/330/345/347 |
| P | Q | 11 | 118/165/192/277/280/304/330/347 |
| P | R | 11 | 26/122/144/304 |
| P | S | 828 | 5/18/26/38/53/88/94/118/122/140/144/165/181/192/193/213/240/277/280/297/303/304/306/330/345 |
| P | T | 57 | 5/18/38/140/165/181/192/280/297/304/306/330/347 |
| P | Y | 2 | 193/240 |
| Q | D | 14 | 175 |
| Q | E | 6 | 22/35/52/172/261 |
| Q | H | 62 | 7/22/23/35/52/57/100/139/147/172/175/180/249/253/361 |
| Q | K | 850 | 6/7/22/23/52/57/136/139/175/235/249/250/261/355/361 |
| Q | L | 14 | 120/139/172/256/267 |
| Q | N | 2 | 361 |
| Q | P | 10 | 6/56/57/100/120/139/147/180 |
| Q | R | 1176 | 35/52/56/172/235/249/250/253/261/355 |
| Q | T | 1 | 7 |
| R | A | 2 | 296 |
| R | C | 1027 | 14/133/148/204/232/258/296/309 |
| R | E | 1 | 349 |
| R | G | 14 | 133/204/232/296/309/349 |
| R | H | 683 | 14/133/148/177/204/232/258/296/309 |
| R | K | 3 | 349 |
| R | L | 9 | 14/133/177/204/232/258/309 |
| R | P | 6 | 80/148/258/296 |
| R | Q | 3 | 80/296 |
| R | S | 29 | 14/177/204/232/258/296/309/349 |
| R | T | 1 | 349 |
| R | Y | 3 | 232/296 |
| S | A | 152 | -4/128/153/311 |
| S | C | 8 | -4/66/154/282/287 |
| S | D | 3 | 129 |
| S | F | 6 | -4/127/166 |
| S | G | 32 | 66/86/129/169/287/324 |
| S | I | 1252 | 86/129/169/236/282/287/324 |
| S | L | 6 | 212/311 |
| S | N | 40 | 86/129/154/169/236/282/287/324 |
| S | P | 14 | -6/-4/127/153/166/212 |
| S | R | 8 | 86/129/236/287 |
| S | T | 90 | -6/-4/66/128/129/153/225/282 |
| S | Y | 3 | -6/166 |
| T | A | 1478 | -8/-3/15/17/55/58/68/70/89/97/105/146/176/247/262/302/305/316/319 |
| T | I | 287 | -16/-15/-8/-3/15/17/42/68/89/97/105/113/146/176/247/262/305/319 |
| T | K | 170 | -15/-3/15/58/105/146/247/302 |
| T | M | 225 | -16/-15/70/187/247/302/316 |
| T | N | 23 | 17/176/226/302 |
| T | P | 42 | -16/-15/-8/-3/15/42/58/90/111/113/226/247/262/302/305/316/319 |
| T | R | 2 | 105/302 |
| T | S | 62 | -16/-8/42/89/90/113/226/247/262/316/319 |
| T | V | 85 | 89/305 |
| V | A | 45 | 12/30/54/72/125/163/191/223/234/278/308/313/326/337/350 |
| V | D | 6 | 326/350 |
| V | E | 6 | 72/191/209/278/313 |
| V | F | 10 | 178/211/326/350 |
| V | G | 72 | 30/32/65/101/125/163/209/211/223/234/278/313/350 |
| V | I | 102 | 32/54/65/125/178/191/209/211/234/278/308/313/326/350 |
| V | L | 101 | 12/30/32/72/121/234/278/298/308/313/337 |
| V | M | 34 | 12/30/72/121/163/191/223/234 |
| W | C | 199 | 93/101/233/271/276/312/354 |
| W | G | 7 | 101/188/271/276/354 |
| W | L | 12 | 93/142/201/260/276/312/354 |
| W | R | 68 | 43/93/101/138/188/201/233/260/271/276/312/354 |
| W | S | 3 | 101/201/354 |
| Y | C | 7 | 40/92/112/150/199/203 |
| Y | D | 14 | 34/40/45/92/112/135/150/203/221 |
| Y | F | 18 | 34/40/203/221/344 |
| Y | H | 16 | 39/45/112/150/221/259/266/325/344 |
| Y | N | 9 | 39/92/135/199/221/325/344 |
| Y | S | 4 | 40/112/199 |
| * | R | 2 | 362 |
| Total number of mutations: 24,766. Number of amino acid insertions: 91 (one to five amino acids added). Number of amino acid deletions: 33 (removed one to four amino acids). Total number of analyzed BlaEC sequences: 2281. SANC (structural alignment-based numbering of class C beta-lactamases). | | | |

**Table S3.** Average amino acid % identity matrix of the least similar BlaEC enzymes

| **Average amino acid identity** | | | | | | | |
| --- | --- | --- | --- | --- | --- | --- | --- |
|  |  |  |  |  |  |  |  |
| **85.8** | **85.9** | **86.1** | **86.2** | **86.4** | **86.5** | **86.6** | **86.8** |
|  |  |  |  |  |  |  |  |
|  | **BlaEC-68** | **BlaEC-1048** | **BlaEC-P1** | **BlaEC-P2** | **BlaEC-P6** | **BlaEC-P71** | **BlaEC-P79** |
| **BlaEC-14** | **86.6** |  |  |  |  |  |  |
| **BlaEC-68** |  |  |  |  |  |  |  |
| **BlaEC-107** | **86.4** |  |  |  | **86.8** |  |  |
| **BlaEC-108** | **86.4** |  |  |  | **86.8** |  |  |
| **BlaEC-116** | **86.1** |  | **86.2** |  | **86.2** |  |  |
| **BlaEC-241** | **86.6** |  |  |  | **86.8** |  |  |
| **BlaEC-275** | **86.1** |  |  |  | **86.5** |  |  |
| **BlaEC-377** | **85.8** | **86.6** | **85.9** | **86.8** | **85.9** |  |  |
| **BlaEC-378** | **86.4** |  |  |  | **86.8** |  |  |
| **BlaEC-379** | **86.4** |  | **86.6** |  | **86.5** |  |  |
| **BlaEC-380** | **86.6** |  |  |  | **86.8** |  |  |
| **BlaEC-381** | **86.4** |  |  |  | **86.8** |  |  |
| **BlaEC-383** | **86.6** |  |  |  |  |  |  |
| **BlaEC-384** | **86.4** |  |  |  |  |  |  |
| **BlaEC-385** | **86.4** |  |  |  | **86.8** |  |  |
| **BlaEC-386** | **86.4** |  |  |  | **86.8** |  |  |
| **BlaEC-387** | **86.6** |  |  |  |  |  |  |
| **BlaEC-388** | **86.1** |  | **86.2** |  | **86.2** |  |  |
| **BlaEC-402** | **86.1** |  | **86.6** |  | **86.5** |  |  |
| **BlaEC-403** | **86.4** |  | **86.6** |  | **86.5** |  |  |
| **BlaEC-404** | **86.4** |  |  |  | **86.5** |  |  |
| **BlaEC-405** | **86.1** |  |  |  | **86.5** |  |  |
| **BlaEC-406** | **86.6** |  |  |  |  |  |  |
| **BlaEC-407** | **86.6** |  |  |  |  |  |  |
| **BlaEC-408** | **85.8** | **86.6** | **86.6** |  | **85.9** | **86.6** | **86.6** |
| **BlaEC-409** | **86.4** |  | **86.6** |  | **86.5** |  |  |
| **BlaEC-410** | **86.4** |  | **86.6** |  | **86.5** |  |  |
| **BlaEC-411** | **86.6** |  |  |  | **86.8** |  |  |
| **BlaEC-412** | **86.4** |  | **86.6** |  | **86.5** |  |  |
| **BlaEC-413** | **86.6** |  |  |  | **86.8** |  |  |
| **BlaEC-414** | **86.1** |  | **86.2** |  | **86.2** |  |  |
| **BlaEC-415** | **86.6** |  |  |  |  |  |  |
| **BlaEC-418** | **86.6** |  |  |  |  |  |  |
| **BlaEC-421** | **86.1** |  |  |  | **86.5** |  |  |
